# Gastric Adenocarcinoma and Proximal Polyposis of the Stomach in a Hispanic Pediatric Patient With APC Gene Variant c.-191T>G

**DOI:** 10.1097/PG9.0000000000000123

**Published:** 2021-09-23

**Authors:** Annette Gawron Roberts, Malgorzata Bujarska, Mislen Bauer, Carole Brathwaite, Liset Pelaez, Jesse Reeves-Garcia

**Affiliations:** From the Departments of Gastroenterology, Medical Education, and Pathology, Nicklaus Children’s Hospital, Miami, Florida.

**Keywords:** gastric polyps, GAPPS, gastric cancer, gastric adenocarcinoma, polyposis, APC, Promoter 1B, hereditary cancer syndrome, polyposis syndromes, APC gene variants, gastric polyposis

## Abstract

Gastric adenocarcinoma and proximal polyposis of the stomach (GAPPS) is a rare gastric polyposis syndrome defined by numerous polyps (>100) in the fundus and body of the stomach with sparing of the lesser curvature and antrum. GAPPS is linked to a variant in the promoter 1B region of the APC gene. These variants carry a high risk of developing gastric adenocarcinoma, which can occur at an early age. We report a case of GAPPS discovered in a 16-year-old Hispanic girl after endoscopy detected extensive fundic gland polyposis. Genetic testing revealed a promoter 1B point mutation of the APC gene, variant c.-191T>G. Although similar variants have been reported (i.e., c.-191T>C, c.-195A>C, c.-192A>G) in association with GAPPS, variant c.-191T>G has not nor has GAPPS ever been described in a Hispanic individual before.

Gastric adenocarcinoma and proximal polyposis of the stomach (GAPPS) is a rare autosomal dominant gastric polyposis syndrome linked to a variant in the promoter 1 B region of the APC gene (1–7). It is defined by the presence of numerous polyps (>100 polyps) in the body and fundus of the stomach with sparing of the antrum, small intestines, and colon (1,2,6,8,9). The sparing of the antrum, colon, and intestines distinguishes it from familial adenomatous polyposis (FAP) syndrome, an autosomal dominant polyposis syndrome characterized by multiple adenomatous polyps affecting the colon and rectum also linked to variants in the APC gene (5,6).

There have been few reported cases of GAPPS in the literature and all reported cases have identified variants in the promoter 1B of the APC gene (2–4,6,9). Li et al. reported 3 different point mutations (c.-195A>C, c.-191T>C, c.-192A>G) linked to GAPPS that affect the expression of the promoter 1 B of the APC gene (6). GAPPS has been identified in people of Australian, white American, white Canadian, Czech, and Japanese descent (2,4,6,9). There has not been a case report or study published to date identifying GAPPS in a Hispanic patient. This report examines the case of a 16-year-old Hispanic girl of Cuban descent diagnosed with GAPPS after endoscopy revealed extensive fundic gland polyposis. Genetic testing identified an APC gene promoter 1B point mutation, variant c.-191T>G.

## CASE REPORT

**FIGURE 1.**
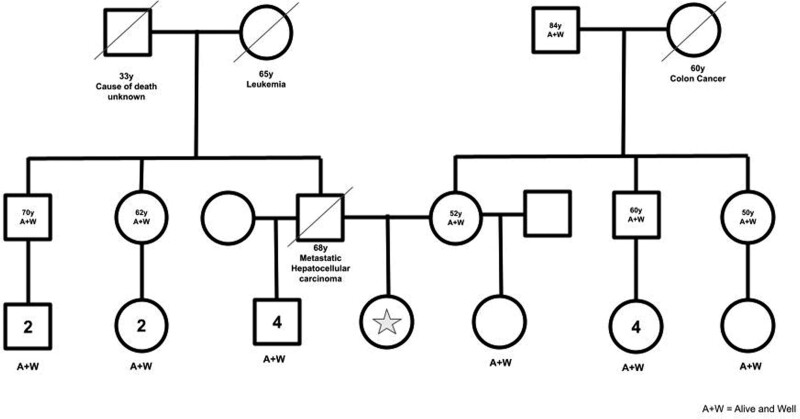
Patient’s family pedigree.

A 16-year-old Hispanic girl of Cuban descent was referred to our gastroenterology clinic following several months of intermittent epigastric pain, dyspepsia, nausea, and vomiting. Her medical history was notable for clinically diagnosed dyspepsia and irregular menses secondary to polycystic ovarian syndrome (BMI in the 55th percentile). Her only home medication was a daily oral contraceptive pill. Notable family history included her father who died of metastatic hepatocellular carcinoma at age 68, a maternal grandmother who died of colon cancer at age 60, and a paternal grandmother who died of leukemia at age 65 (Fig. 1).

**FIGURE 2.**
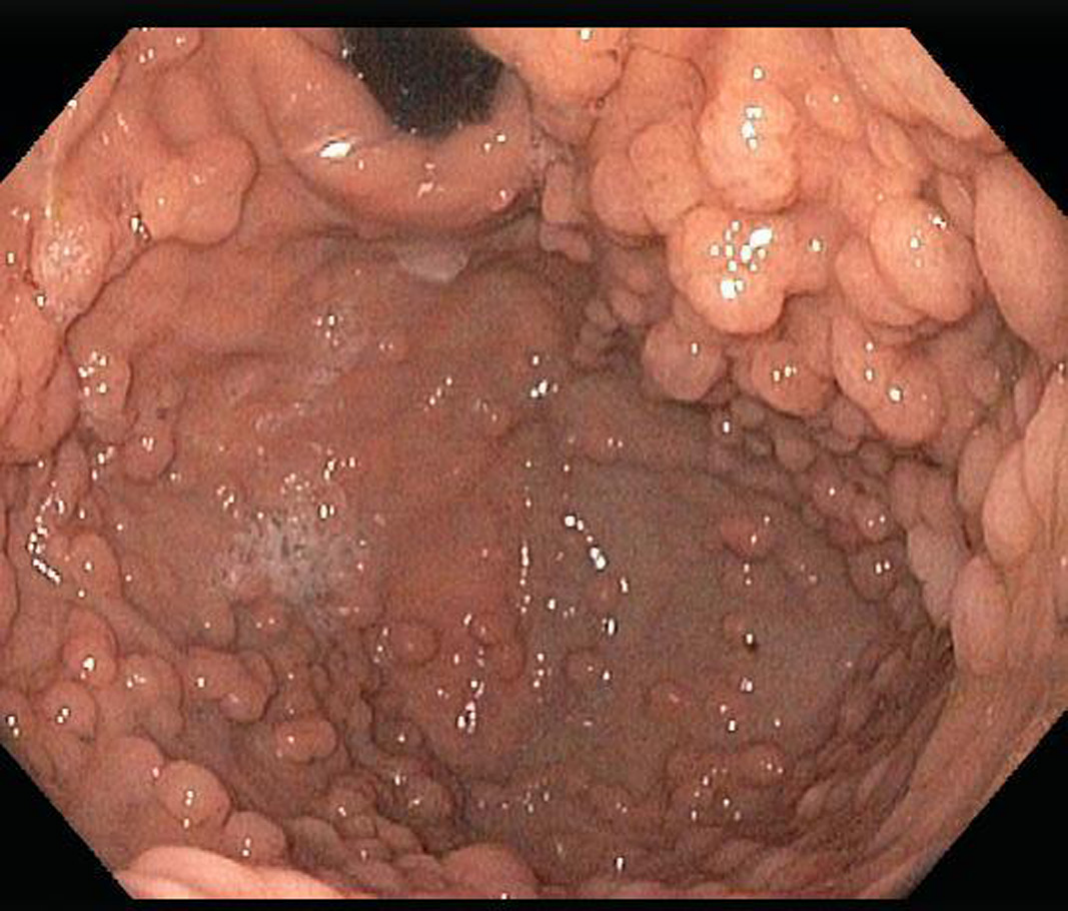
Endoscopy illustrating over 150 fundic gland polyps.

**FIGURE 3.**
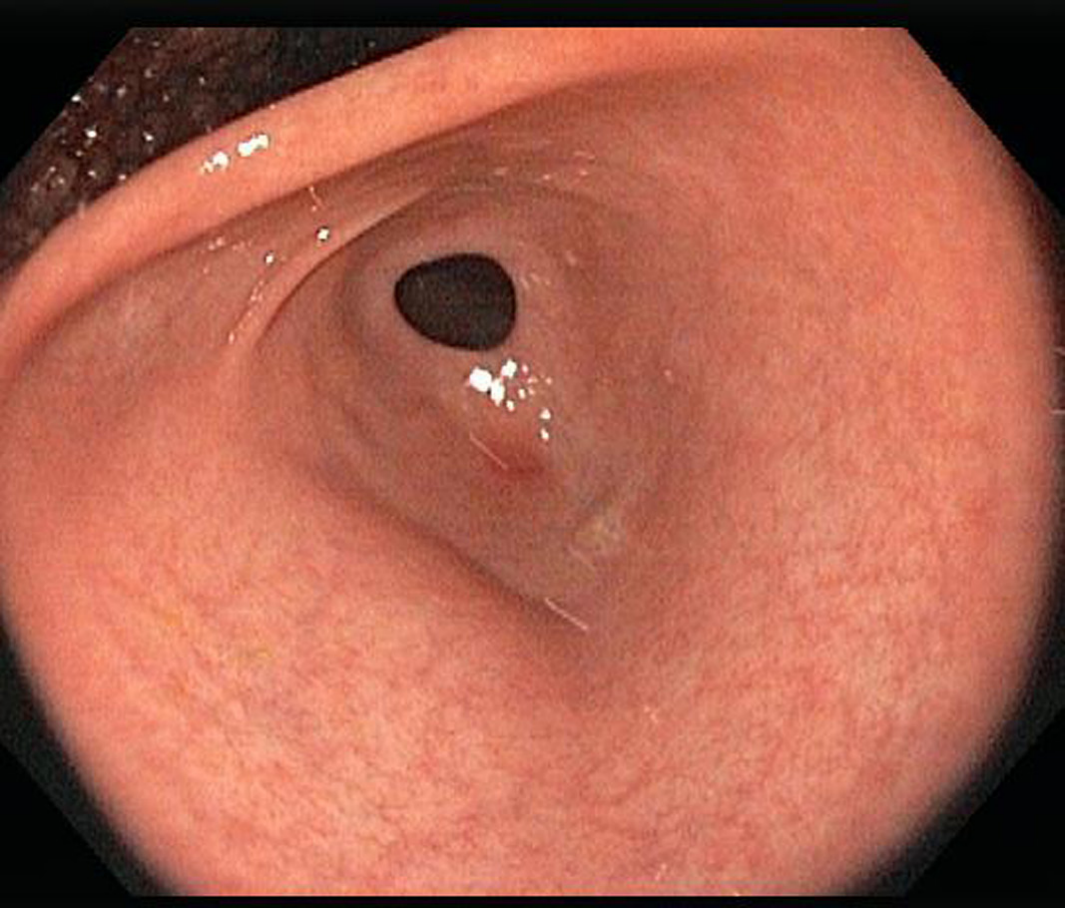
No polyps were identified on endoscopy in the antrum and pylorus.

**FIGURE 4.**
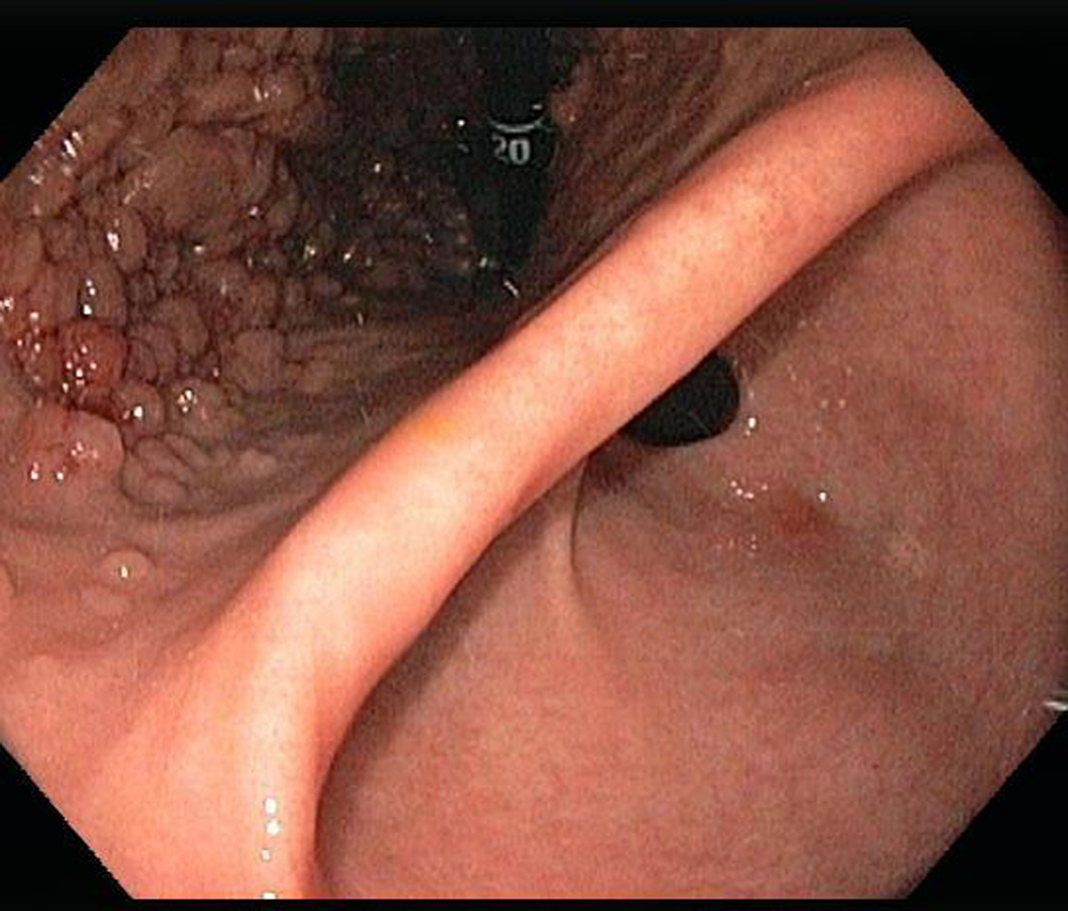
Endoscopy illustrating numerous fundic gland polyps with sparing of the antrum and pylorus..

**FIGURE 5.**
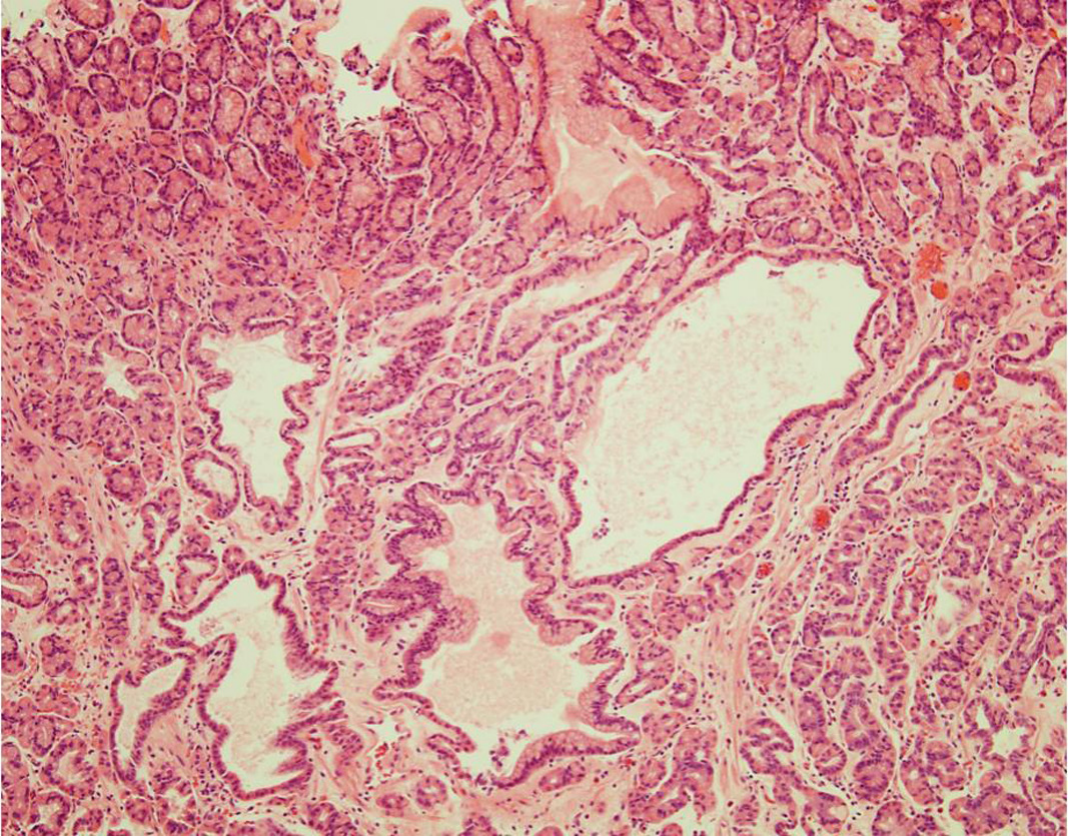
Fundic gland polyp biopsy specimen showing a distorted glandular architecture composed of irregular and cystically dilated glands with stellate configuration.

The patient’s physical examination and vitals were found to be unremarkable during the initial visit. Laboratory workup with complete blood count, urinalysis, and comprehensive metabolic panel were all within normal limits except a lipid panel with mildly elevated cholesterol level. Stool culture, stool *Helicobacter pylori*, and stool ovum and parasite testing were all negative. The patient was sent for an endoscopy which detected over 150 fundic gland polyps (Fig. 2). No polyps were seen in the antrum, pylorus, or small bowel (Figs. 3 and 4). The esophagus was unremarkable. Four polyps were removed in toto by polypectomy and sent to pathology. Microscopic examination of the fundic gland polyps showed distorted glandular architecture composed of irregular and cystically dilated glands with stellate configuration lined by normal gastric body-type epithelium (Fig. 5). No atypia or dysplasia was reported. One week later, the patient returned for a complete workup with colonoscopy, capsule endoscopy, and repeat endoscopy. This time, 2 polyps were removed in toto by polypectomy with similar findings on pathology. The mucosa of the esophagus, antrum, duodenum, and colon was unremarkable. Colonoscopy did not detect any polyps in the colon and biopsies showed unremarkable colonic mucosa without evidence of aberrant crypt foci. Video-Capsule Endoscopy revealed gastric fundus polyposis and lymphoid hyperplasia of the terminal ileum. No small bowel polyps were identified.

The patient was referred for full genetic workup and genetic counseling to a clinical geneticist at our institution with experience in the clinical management and genetic workup of patients with suspected underlying hereditary cancer syndromes. Given the extent of polyposis in our patient with a strong family history of malignancies, our geneticist selected the OncoGeneDx Colorectal Panel by GeneDx, an OPKO Health company located in Gaithersburg, MD. The rationale for the selection of this gene panel was its ability to examine 20 different genes involved in hereditary colorectal cancer and other gastrointestinal cancers. Buccal mucosa DNA was used for this panel and informed consent was obtained from the patient’s mother before the samples were collected.

Next-generation sequencing using the OncoGeneDx Colorectal Cancer Panel identified a variant in the promoter 1B region of the APC gene, c.-191T>G, a nucleotide substitution 191 base pairs upstream of the ATG translational start site in the 5′ untranslated region. This variant was considered “likely pathogenic” in the genetic report. The patient was also positive for a POLE gene variant, c.2510T>C, at the cDNA level and p.Phe837Ser at the protein level. This variant was considered a variant of uncertain significance. The patient was negative for *PTEN*, *STK11*, *BMPR1A*, *SMAD4*, *ATM*, *AXIN2*, *CDH1*, *CHEK2*, *EPCAM*, *MLH1*, *MSH2*, *MSH6*, *MUTYH*, *NTHL1*, *PMS2*, *POLD1*, *SCG5/GREM1*, and *TP53* gene variants. Genetic testing was recommended to the patient’s mother and relatives. The mother’s genetic testing did not reveal any *APC* gene mutations, but she did share the same variant of uncertain significance on the *POLE* gene. The patient’s father’s other 4 offspring all of whom live in Cuba were contacted and informed of their risks.

We advised our patient to undergo a prophylactic total gastrectomy. At first, the family refused the procedure. The mother later sought out the National Institute of Health who became interested in her case and also recommended total gastrectomy. The patient had a prophylactic total gastrectomy in June of 2021 and tolerated the procedure well. Given the novelty of our variant, c.-191T>G, the variant was reported by our team to the LOVD3 database for APC mutations.

## DISCUSSION

GAPPS is a rare autosomal dominant gastric polyposis syndrome linked to a variant in the promoter 1B region of the *APC* gene (1–7). It is defined by the presence of numerous polyps (>100 polyps) in the body and fundus of the stomach with sparing of the antrum, small intestines and colon (1,2,6,8,9). The sparing of the antrum, colon, and intestines distinguishes it from FAP syndrome, an autosomal dominant polyposis syndrome characterized by multiple adenomatous polyps affecting the colon and rectum also linked to variants in the APC gene (5,6). FAP may occasionally present with extensive gastric fundic gland polyposis especially in individuals with large deletions in the promoter 1B (5,6,10,11).

There have been few reported cases of GAPPS in the literature and all reported cases have identified variants in the promoter 1B of the APC gene (2–4,6,9). Li et al. reported 3 different point mutations (c.-195A>C, c.-191T>C, c.-192A>G) that affect the expression of the promoter 1B of APC by interruption of the Ying Yang 1 (YY1) binding motif (6). Deletions in promoter 1B have been observed in some cases of FAP, however, point mutations in promoter 1B rarely lead to FAP and are more closely associated with GAPPS (6). The APC gene promoter 1B is known to be highly transcribed in the gastric mucosa compared with the intestinal mucosa (4,6). Furthermore, it is believed that the colon and intestines are protected by the promoter 1A of the gene, which accounts for the sparing of the intestinal mucosa in patients with GAPPS (4,6).

The age of onset is highly variable among individuals with GAPPS and is likely influenced by multiple factors including genetics, environment, and lifestyle habits (4). In those with extensive polyposis in the setting of a promoter 1B APC variant, the risk of developing gastric adenocarcinoma is significant and ranges between 12% and 25% based on current studies (2–4). In a study done by Foretova et al. at the Masaryk Memorial Cancer Institute (MMCI) in Brno, Czech Republic, 6 out of 24 individuals carrying the APC gene promoter 1B point mutation (variant c.-191T>C) were diagnosed with gastric cancer (4). The earliest age at diagnosis reported in their study was 22 years (4). In the patients observed by Repak et al., 1 female (age 26) developed poorly differentiated adenocarcinoma with metastasis despite endoscopic surveillance with multiple biopsies every 18–24 months (2). She died shortly after beginning palliative chemotherapy (2).

The transition from dysplasia to gastric cancer can be rapid and endoscopic surveillance often misses tumors or dysplastic polyps making endoscopic surveillance a poor option for early cancer detection (4). Therefore, the MMCI recommends prophylactic total gastrectomy with D2 lymphadenectomy consistent with the standard of care for stomach malignancies to all patients identified with extensive gastric polyposis secondary to GAPPS (4). Currently, nongastric manifestations of GAPPS are poorly defined in literature (7). Despite relative sparing of the antrum, intestines, and colon, colonic polyps have been reported in literature in patients with the GAPPS phenotype (7). As a result, Martin et al. advocate for colonoscopic surveillance in the routine management of patients with confirmed GAPPS (5). Furthermore, genetic testing should be recommended to the relatives of a GAPPS affected individual according to the National Comprehensive Cancer Network Clinical Practice Guidelines for gastric cancer. The MMCI currently recommends genetic testing to all relatives of patients with confirmed GAPPS (4).

GAPPS has been identified in people of Australian, white American, white Canadian, Czech, and Japanese descent (2,4,6,9). To date, there have been few studies reporting GAPPS none of which describe GAPPS in a person of Hispanic descent. Furthermore, c.-191T>C, c.-192A>G, and c.-195A>C point mutations in the promoter 1B of the APC gene have been identified, but the gene variant exhibited in our patient, c.-191T>G, has not (4,7). The APC variant c.-191T>G is also unreported in multiple genetic databases making it unique. In Varsome, it is classified as a “Variant of Unknown Significance.” This case introduces a new variant in a unique patient population and highlights both the importance of early detection of GAPPS with endoscopy and early intervention.

## ACKNOWLEDGMENTS

The mother provided consent for publication of the details of this case.

After taking into consideration the risk of gastric cancer associated with promoter 1B APC gene variants and the limitations of endoscopic surveillance, a prophylactic gastrectomy was recommended to our patient who ultimately agreed to the procedure.
